# Enhancing Cross-Cultural Intergenerational Capacity in Social Capital

**DOI:** 10.1093/geroni/igaf122.861

**Published:** 2025-12-31

**Authors:** Daniel W L Lai, Hamza Saghir Aslam, Hazy H Y Lee, Alice C Wong

**Affiliations:** Hong Kong Baptist University, Kowloon, China (People’s Republic); Hong Kong Baptist University Department of Sports and Health Sciences, Kowloon, China (People’s Republic); Hong Kong Baptist University, Kowloon, Hong Kong, China (People’s Republic); Hong Kong Baptist University, Kowloon, Hong Kong, China (People’s Republic)

## Abstract

This research presents the impactful findings of the “Enhancing Cross-Cultural Intergenerational Capacity in Social Capital” project, which successfully connected university students with older South Asians in Hong Kong, addressing critical issues of social isolation and cultural misunderstanding. Older South Asians often encounter barriers to community engagement and resource access. This initiative aimed to enhance students’ cultural competency while empowering older adults, fostering stronger community ties and promoting inclusivity. The project sought to cultivate cross-cultural communication skills among students, facilitate meaningful intergenerational interactions, and co-create culturally relevant services tailored to the unique needs of older South Asians. Through cultural competency training and collaborative co-creation workshops, students worked alongside community partners and older adults to design services that resonate with the community. Engaging over 50 participants, the project led to transformative outcomes: participants reported a 30% increase in cultural understanding and improved social connectivity, while students experienced a 40% boost in confidence in their service delivery. Qualitative feedback highlighted powerful personal stories of connection and growth, illustrating the profound impact on both students and older adults. These findings underscore the essential role of inclusive service-learning initiatives in fostering social capital and intergenerational solidarity, offering valuable insights for future community engagement practices and research.

